# Transient Budd–Chiari syndrome secondary to blunt traumatic bile fistula: A case report

**DOI:** 10.3389/fsurg.2022.951514

**Published:** 2022-09-01

**Authors:** Qimin Ma, Kai Cao, Pengfei Luo, Xiaobin Liu, Tuo Shen, Yusong Wang, Feng Zhu

**Affiliations:** ^1^ Burns & Trauma ICU, The First Affiliated Hospital, Naval Medical University, Shanghai, China; ^2^ Department of Radiology, The First Affiliated Hospital, Naval Medical University, Shanghai, China

**Keywords:** Budd–Chiari syndrome, trauma, inferior vena cava, bile fistula, case report

## Abstract

Budd–Chiari syndrome (BCS) is rarely caused by trauma. We reported a case of transient and secondary BCS post polytrauma that resulting from massive perihepatic and abdominal fluid and compressed liver, causing stenosis of the inferior vena cava and hepatic veins. This was a special BCS case related to but not directly caused by trauma. With conservative management and active surgical procedures, the patient recovered well.

## Introduction

Budd–Chiari syndrome (BCS) comprises a heterogeneous group of conditions characterized by partial or complete hepatic venous outflow obstruction involving one or more hepatic veins (HVs), the inferior vena cava (IVC), or the right atrium. The most common causes include a hypercoagulable or prothrombotic state, extension of the thrombus, a direct mass effect caused by liver lesions including liver tumors and abscesses, and congenital development abnormality of IVC (diaphragm formation, stenosis, and atresia). BCS is rarely caused by trauma that influences mortality because of massive bleeding and the difficulty associated with its repair. Common treatments for BCS include anticoagulation therapy, transjugular intrahepatic portosystemic shunting, surgical shunting, and liver transplantation. The risks and benefits of living donor liver transplantation have also been the focus of recent attention (1, 2). In the present case, BCS, associated with polytrauma, actually resulted from compression of partially perihepatic encapsulated fluid and abdominal fluid because of traumatic bile fistula.

## Case presentation

**Figure 1:**
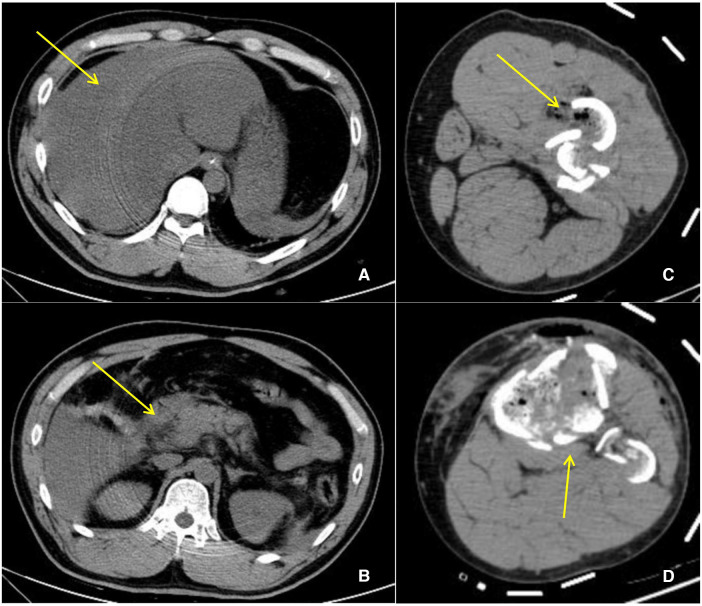
CT manifestation post polytrauma. (**A**) Liver rupture, perihepatic hemorrhage (yellow arrow). (**B**) Exudation around the pancreatic head. (**C**) Fracture of the left femoral backbone. (**D**) Fracture of the left tibia.

**Figure 2:**
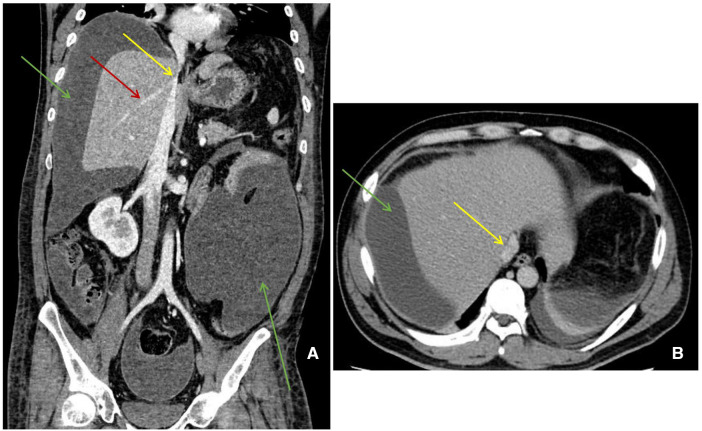
Abdominal enhanced CT manifestation. (**A**) (Coronal) a large amount of perihepatic and abdominal fluid (green arrow) and stenosis of IVC (yellow arrow) and hepatic veins (red arrow). (**B**) (Horizontal) a large amount of perihepatic fluid (green arrow) and stenosis of IVC (yellow arrow).

**Figure 3:**
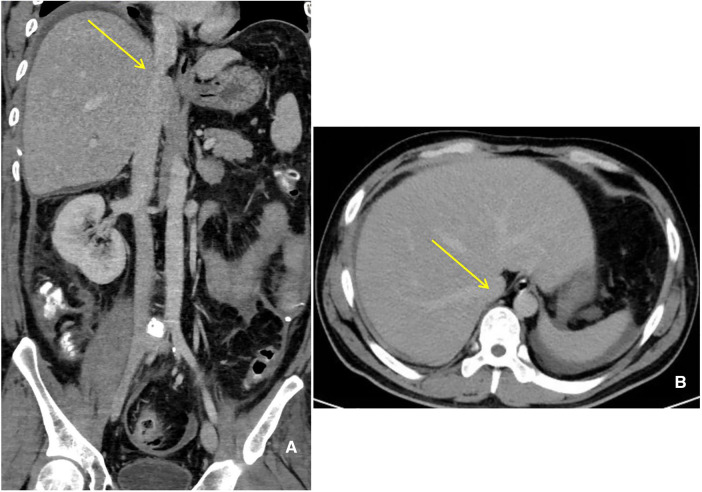
Significant improved on the compressed IVC (yellow arrow). (**A**) Coronal. (**B**) Horizontal.

A 35-year-old man with polytrauma was transferred to our Burns & Trauma ICU on September 1, 2021. He was accidentally smashed in the left thigh by a trunk while sawing the tree and was then picked by other dropped trunks to a height of 3 m and hurt severely with pain. In the Emergency Department, computed tomography (CT) showed liver rupture, perihepatic hemorrhage, exudation around the pancreatic head, abdominal and pelvic effusion, fracture of the left femoral backbone, and fracture of the left tibia (Figures 1A–D). After comprehensive conservative treatment including antishock, transfusion, hemostatic resuscitation, and anti-infection treatment, the patient was stable gradually. Since September 24, ascites have increased progressively accompanied by nausea and vomiting, abdominal distension, and jaundice. To make a diagnosis, abdominal enhanced CT and diagnostic abdominocentesis were performed. Abdominal enhanced CT showed a large amount of perihepatic and abdominal fluid (partially encapsulated) and compressed liver, causing stenosis of IVC and HVs (Figures 2A,B). Ascites examination revealed a significant increase in total bilirubin and direct bilirubin with normal amylase. We considered a presumptive diagnosis: bile fistula post-polytraumatic liver rupture, perihepatic effusion, ascites, and secondary BCS. The abdominal distension and ascites were relieved after intraperitoneal centesis and drainage, and abdominal enhanced CT on September 29 showed a significant improvement of the compressed IVC and hepatic veins (Figures 3A,B).

**Figure 4:**
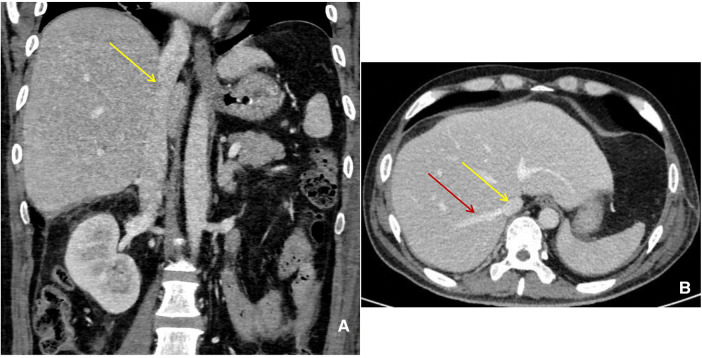
Abdominal enhanced CT manifestation. (**A**) (Coronal) IVC (yellow arrow) returned to be normal. (**B**) (Horizontal) IVC (yellow arrow) and hepatic veins (red arrow) returned to be normal.

The following day, the patient still had intermittent abdominal distension, abdominal pain, and fever with slightly elevated inflammatory indicators (PCT, CPR, WBC, etc.) as well as jaundice. To find the cause of bile fistula, a laparotomy was performed on October 7. Rupture of the distal choledochusal near the pancreatic duct, extensive intestinal adhesion, and encapsulated effusions were found in the surgery. Choledochal “T” tube drainage + enteroclysis + peritoneal effusion removal was performed. Abdominal distension, abdominal pain and fever, and inflammatory indicators significantly improved, and ascites disappeared postsurgery. Abdominal enhanced CT on November 8 showed that IVC and hepatic veins returned to be normal (Figures 4A,B).

## Discussion

BCS is defined as “the obstruction of hepatic venous outflow that can be located from the small hepatic venules up to the entrance of IVC into the right atrium, if a right heart failure or constrictive pericarditis has been excluded” (3). Since the first description by Dr. Budd and Dr. Chiari, it took two centuries to coin such a definition (4, 5). BCS is common in hepatic vein/IVC thrombosis, tumor invasion or compression, and IVC congenital developmental abnormalities (diaphragm formation, stenosis, closure) but is rarely caused by trauma. BCS directly caused by trauma is often fatal because of massive bleeding and the difficulty associated with its repair. In this case, although polytrauma was the cause, it did not directly lead to IVC thrombosis or massive bleeding. Perihepatic and abdominal massive accumulation of fluid (partially encapsulated) caused by traumatic biliary fistula compressed liver and HVs/IVC, which caused BCS finally. Whether traumatic biliary fistula is primary or secondary was unclear. Perhaps, this was a nonclassic trauma-related BCS. BCS clinical manifestations greatly varied, including lack of appetite, nausea and vomiting, abdominal distension, ascites, large liver, jaundice, fatigue, scrotum, and lower limb edema (6, 7). In this patient, the symptoms and signs were less typical, probably because of the gradual ascites and compression. This presents difficulties in the clinical and differential diagnoses even if the signs of the BCS could be seen on enhanced CT. We finally found *via* surgery that the true cause was a biliary fistula.

Emergent surgical management of BCS including primary repair, patch repair, or reconstruction with various grafts is used to manage BCS caused by trauma, but the optimal strategy for surgery remains controversial. There are many causes of BCS, which need to be identified by the clinician in the context of the medical history and examination. In the past, a patient with liver disease had BCS due to a giant hydatid cyst, which was effectively relieved by catheterization with a drainage catheter in the cystic cavity (8). In these BCS patients, venous compression relief is an effective way to protect liver function (9). Furthermore, trauma did not directly lead to injury and rupture of IVC/HVs. BCS was due to the compression of the liver, IVC, and HVs caused by biliary fistula and ascites. Thus, choledochal “T” tube drainage + enterolysis + peritoneal effusion removal could reduce abdominal stimulation and ascites, reduce compression, and finally alleviate BCS.

Overall, this patient had massive perihepatic and abdominal encapsulated effusion due to liver rupture and bile fistula. Then, effusion extrusion of the liver caused severe stenosis of IVC/HVs, resulting in clinical transient secondary BCS. In turn, secondary BCS aggravated ascites and clinical symptoms. After removing the inclusion effusion and reducing liver extrusion via surgery, the symptoms and signs improved and disappeared. We fully recorded the development and management of this transient secondary BCS case. At the same time, we also realize that this is a nonclassic trauma-related BCS case, and ascites clearance and repair of bile fistula are keys for the management of transient secondary BCS.

## Data Availability

The original contributions presented in the study are included in the article/Supplementary Material, further inquiries can be directed to the corresponding author.
